# Mutations in *RPSA* and *NKX2‐3* link development of the spleen and intestinal vasculature

**DOI:** 10.1002/humu.23909

**Published:** 2019-09-23

**Authors:** Chantal Kerkhofs, Servi J. C. Stevens, Saul N. Faust, William Rae, Anthony P. Williams, Peter Wurm, Rune Østern, Paul Fockens, Christiane Würfel, Martin Laass, Freddy Kokke, Alexander P. A. Stegmann, Han G. Brunner

**Affiliations:** ^1^ Department of Clinical Genetics and GROW‐School for Oncology and Developmental Biology Maastricht University Medical Center Maastricht The Netherlands; ^2^ NIHR Southampton Clinical Research Facility and NIHR Southampton Biomedical Research Centre, University of Southampton Faculty of Medicine and University Hospital Southampton NHS Foundation Trust Southampton UK; ^3^ Departments of Immunology and Paediatric Immunology and Infectious Diseases University Hospital Southampton UK; ^4^ Department of Gastroenterology University Hospitals of Leicester, NHS Trust Leicester UK; ^5^ Department of Pathology and Medical Genetics St. Olavs Hospital Trondheim Norway; ^6^ Department of Gastrointestinal diseases Academic Medical Center Amsterdam The Netherlands; ^7^ Department of Pediatrics University Hospital Dresden Dresden Germany; ^8^ Department of Pediatric Gastroenterology Maastricht University Medical Center Maastricht The Netherlands; ^9^ Department of Human Genetics, Donders Institute for Brain, Cognition, and Behaviour Radboud University Medical Center Nijmegen The Netherlands

**Keywords:** asplenia, homeobox gene, intestinal varices, *NKX2‐3*, *RPSA*, whole‐exome sequencing

## Abstract

Idiopathic intestinal varicosis is a developmental disorder defined by dilated and convoluted submucosal veins in the colon or small bowel. A limited number of families with idiopathic intestinal varices has been reported, but the genetic cause has not yet been identified. We performed whole‐exome and targeted Sanger sequencing of candidate genes in five intestinal varicosis families. In four families, mutations in the *RPSA* gene were found, a gene previously linked to congenital asplenia. Individuals in these pedigrees had intestinal varicose veins and angiodysplasia, often in combination with asplenia. In a further four‐generation pedigree that only showed intestinal varicosities, the *RPSA* gene was normal. Instead, a nonsense mutation in the homeobox gene *NKX2‐3* was detected which cosegregated with the disease in this large family with a LOD (logarithm of the odds) score of 3.3. *NKX2‐3* is a component of a molecular pathway underlying spleen and gut vasculature development in mice. Our results provide a molecular basis for familial idiopathic intestinal varices. We provide evidence for a relationship between the molecular pathways underlying the development of the spleen and intestinal mucosal vasculature that is conserved between humans and mice. We propose that clinical management of intestinal varices, should include assessment of a functional spleen.

## 1 INTRODUCTION

The presence of dilated and convoluted submucosal veins in the colon or small bowel referred to as intestinal varices is a rare clinical entity with a poorly understood etiology (Speicher, Keegan, & Kirk, 2014). Intestinal varices may cause recurrent bleeding of the lower gastrointestinal tract or may be noticed in an asymptomatic individual upon colonoscopy. The most prevalent cause of varices in the digestive tract is portal hypertension. About one‐quarter of reported cases of varices coli are idiopathic, and 30 % of these are familial. (Han et al., 2006) Thus far, at least 12 families with idiopathic intestinal varices have been reported in the literature but no genetic cause has been identified. (Atin, Sabas, Cotano, Madariaga, & Galan, 1993; Beermann, Lagaay, van Nouhuys, & Overbosch, 1988; Bernardini et al., 1998; Boland, Leonard, Saunders, & Bursey, 2014; el‐Dosoky, Reeders, Dol, & Tytgat, 1994; Hawkey, Amar, Daintith, & Toghill, 1985; Iredale, Ridings, McGinn, & Arthur, 1992; Kori, Keter, Grunshpan, Zimmerman, & Ackerman, 2000; Morini, Caruso, & De Angelis, 1993; Solis‐Herruzo, 1977; Zaman, Bebb, Dunlop, Jobling, & Teahon, 2008). In some families, siblings are affected (Atin et al., 1993; Boland et al., 2014; Kori et al., 2000), suggestive of autosomal recessive inheritance, whereas in other families, the intestinal varices occur in two generations (el‐Dosoky et al., 1994; Solis‐Herruzo, 1977; Zaman et al., 2008), consistent with autosomal dominant inheritance. Here, we describe five families with autosomal dominant intestinal varices, and identify mutations in the *RPSA* gene or *NKX2‐3* gene as a genetic cause. Although evidence for a direct interaction between RPSA‐ and NKX2‐3‐related pathways is currently lacking, we show evidence from multiple sources that link the molecular programs underlying the development of the intestinal vasculature and spleen, both in humans and mice.

## 2 MATERIALS AND METHODS

### 2.1 Whole‐exome sequencing

After obtaining written informed consent, whole‐exome sequencing was done using DNA isolated from blood, as described previously (Lelieveld et al., 2016). Briefly, exome capture was done using the Agilent SureSelect v4 Kit (Agilent, Santa Clara, CA). Exome libraries were sequenced on an Illumina HiSeq instrument (Illumina, San Diego, CA) with 101‐bp paired‐end reads at a median coverage of 75× and with >95% of exons having coverage >30×. Sequence reads were aligned to the hg19 reference genome using BWA version 0.5.9‐r16. Variants were subsequently called by the GATK unified genotyper, version 3.2‐2 and annotated using a custom diagnostic annotation pipeline. Variants were filtered for having less than 1% frequency in dbSNP, having <1% frequency in our in‐house database and having <1% frequency in the “Exome Aggregation Consortium” (ExAC) database (http://www.exac.broadinstitute.org).

### 2.2 Sanger sequencing

Sanger sequencing was done according to standard procedures, using M13 tailed forward and reverse primers for each exon of the *RPSA* gene or *NKX2‐3* gene. Primer sequences are given in Table S1.

Patients gave informed consent for the genetic studies, which were done in a routine diagnostic setting, and for inclusion of the data in this manuscript.

Variants detected in the *NKX2‐3* gene and *RPSA* gene were reported to the Global Variome shared Leiden Open Variation Database at https://databases.lovd.nl/shared/genes/RPSA and https://databases.lovd.nl/shared/genes/NKX2-3, respectively.

## 3 RESULTS

### 3.1 Patients

**Figure 1:**
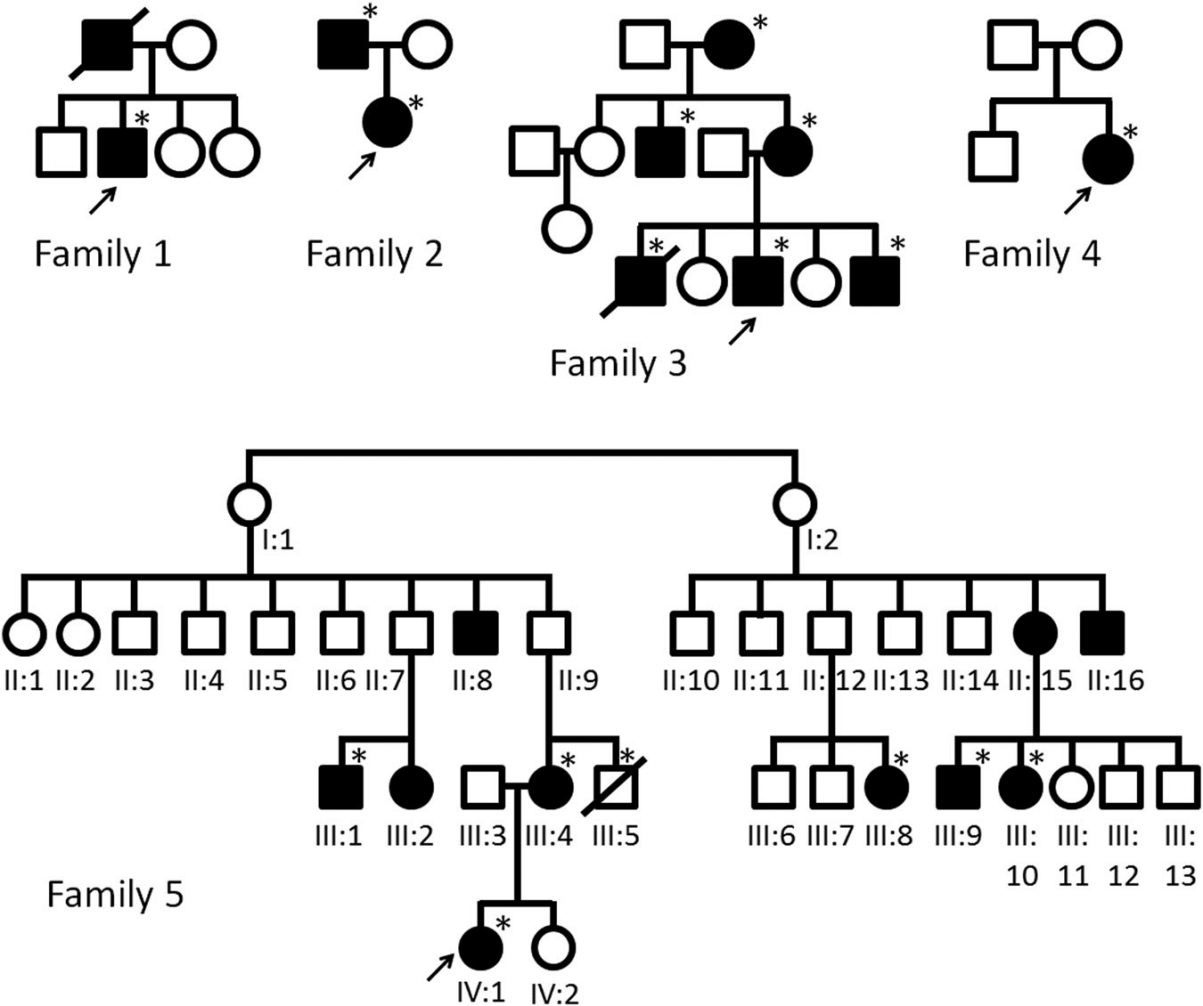
Pedigrees of Families 1–5 described in this study. The proband in each family is indicated by an arrow. Filled (black) symbols indicate intestinal varices. An asterisk indicates the presence of a heterozygous variant in either the *RPSA* or *NKX2‐3,* that is, in Family 1, a c.223dup (p.(Ser75Lysfs*36)) variant in *RPSA*, in Family 2, a c.252G>C (p.(Gln84His)) variant in *RPSA,* in Family 3, a c.538C>G (p.(Arg180Gly)) variant in *RPSA*, in Family 4, a c.542A>T (p.(Glu181Val)) variant in *RPSA* and in Family 5, a c.268del (p.(Gln90Argfs*25)) variant in *NKX2‐3*

**Figure 2:**
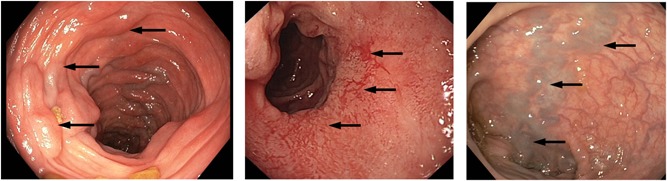
Macroscopical presentation of intestinal varices as assessed by colonoscopy. Arrows indicate examples of intestinal varices as observed in the proband of Families 1, 3, and 5 (from left to right, respectively)

The index patient in Family 1 presented with anemia from the age of 10, which required blood transfusions on several occasions. Colonoscopy demonstrated intestinal varices in the colon and to a lesser extent in the small bowel. At age 48, he developed bacterial meningitis. Abdominal imaging showed an absence of the spleen. The index patient in Family 2 had been hospitalized with bacterial meningitis at the age of 3 years. She developed severe anemia from the age of 27 years. She was hospitalized repeatedly for recurrent gastrointestinal bleedings from varicose veins in the ascending and transverse colon. There was involvement of the small bowel with varicosities in the jejunum. On abdominal imaging, the spleen was described as “multiseptated” or “fragmented”, possibly representing polysplenia. Family 3 was reported previously as presenting with idiopathic congenital asplenia (Bolze et al., 2013). We re‐evaluated the clinical data for some of the individuals from this family, who were known to have ectatic blood vessels in their intestines. The index patient presented at 17 years of age with severe fatigue and anaemia managed with repeated transfusions as no cause could be identified at that time. Asplenia was detected on an abdominal computed tomography scan. At age 24, an exploratory laparotomy identified abnormally dilated vessels in the wall of the distal duodenum. Since then, the patient has undergone repeated argon laser cauterization of duodenal blood vessels approximately every 6 months as they are inoperable. Family 4 has been reported previously (Wurfel et al., 2011). The index patient had a history of iron‐deficiency anemia necessitating repeated erythrocyte transfusion from the age of 2 years. At 16 years of age, gastroduodenoscopy and capsule endoscopy revealed distorted telangiectatic vessels in the stomach and numerous angiodysplastic lesions in the duodenum and jejunum. A number of bleeding lesions were treated by argon plasma coagulation. Abdominal imaging confirmed asplenia. The index patient of Family 5 (patient IV:1 in Figure 1) had recurrent episodes of rectal bleeding for which she first underwent a colonoscopy and upper gastrointestinal endoscopy at age 7 years. This failed to demonstrate a source for the intestinal blood loss. Because of recurring rectal bleeding she underwent a second colonoscopy at age 10, which showed prominent, distended blood vessels in the sigmoid (see Figure 2). At the age of 13 years, massive bleeding occurred, requiring transfusion of packed red blood cells. Abdominal imaging documented a morphologically normal spleen. Pedigrees of Families 1–5 are shown in Figure 1. Extended case reports are given in Supporting Information methods.

### 3.2 Detection of pathogenic variants in the *RPSA* gene or *NKX2‐3* gene

**Figure 3:**
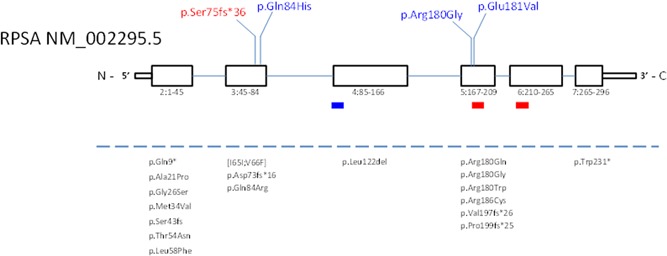
Schematic representation of the *RPSA* gene (NM_002295.5) with published exonic nonsynonymous RPSA mutations. Exons: squares, coding exons 2–7; introns: lines. Below exons: exon and amino acid numbering. Red rectangles: proposed laminin‐binding sites at aa.161–180 and aa.205–229 and blue rectangle: predicted transmembrane domain at aa.86–101. Above the gene schematic: novel mutations identified in this publication. Below dotted line: previously published mutations (Bolze et al., 2018, 2013)

Heterozygous likely pathogenic variants in the *RPSA* gene were detected in Families 1–4 by exome sequencing followed by confirmation with Sanger sequencing and/or by targeted Sanger sequencing (Figures 1 and 3 and Supporting Information data). No variants in the *NKX2‐3* gene were found in these families. In Family 1, a heterozygous c.223dup (p.(Ser75Lysfs*36)) frameshift variant in exon 3 of the *RPSA* gene (NM_002295.5) was found resulting in a premature termination codon in the *RPSA* transcript and most likely leading to haploinsufficiency. The material of the deceased and similarly affected father or of other family members was not available for testing. In Family 2, a c.252G>C (p.(Gln84His)) missense variant was detected in both the affected father and daughter, which affects an evolutionairy conserved amino acid residue in the RPSA protein (conserved up to *Saccharomyces cerevisiae*). A different substitution of the same amino acid residue (p.Gly84Arg) was previously reported in a family with apparently isolated asplenia (Bolze et al., 2013). In Family 3, a c.538C>G (p.(Arg180Gly)) amino acid substitution was found, which was previously discovered and described in the context of a study on isolated congenital asplenia (Bolze et al., 2013). In Family 4, a c.542A>T (p.(Glu181Val)) variant was detected in *RPSA*, affecting an evolutionarily conserved amino acid residue (conserved up to *S. cerevisiae*). None of these *RPSA* variants are present in control individuals from the GnomAD database (http://gnomad.broadinstitute.org).

In Family 5 the whole‐exome sequencing data of two distantly related affected relatives were compared (i.e., individuals IV:1 and III:9; see pedigree in Figure 1), using the filter settings detailed above. Among the shared variants (see Table S2), a heterozygous c.268del (p.(Gln90Argfs*25)) variant in the *NKX2‐3* gene (NM_145285.2) was detected thereby, and subsequently confirmed by Sanger sequencing in these two individuals. Given the absence of this variant in the ExAC database and the fact the variant presumably leads to loss‐of‐function (LoF), other family members were investigated. In individuals, III:4, III:5, III:1, III:8, and III:10 from Family 5, the presence of this frameshift variant in the *NKX2‐3* gene was confirmed by targeted Sanger sequencing. Between these two family branches, there are obligate carriers of this variant. The single‐nucleotide deletion causes a frameshift resulting in a premature termination codon in the *NKX2‐3* transcript, probably leading to haploinsufficiency. The variant cosegregated with the intestinal varices in this family, resulting in a LOD (logarithm of the odds) score of 3.3, indicating that the probability that this particular variant is shared in the five affected family members solely by chance is <1 in 1,000. *NKX2‐3* is predicted to be highly intolerant to LoF variation, as indicated by the absence of LoF variants in the gene in the ExAC database (Lek et al., 2016). The gene has a probability of loss‐of‐function intolerance (pLI) of 0.95, which is very high for such a small gene and an “observed over expected ratio for LoF variants of 0.00 (http://gnomad.broadinstitute.org/gene/ENSG00000119919; Lek et al., 2016). The Database of Genomic Variants currently lists no copy‐number losses or gains for the *NKX2‐3* gene, further indicating the gene to be intolerant to dosage variation.

### 3.3 Genotype–phenotype correlation

The families with *RPSA* variants (Families 1–4) all had a combination of asplenia and intestinal varices. In Family 5, several individuals presented with rectal bleeding. The clinical presentation ranged from mild to severe intestinal bleeding requiring surgical intervention (Supporting Information case reports). However, some obligate carriers of the mutation (Family 5, II:9, II:12) have no known history of rectal bleeding or anaemia, suggesting variable involvement, and possibly reduced penetrance. Such individuals might still have asymptomatic intestinal varices as there was no clinical indication to perform a colonoscopy in them. In Family 5, only intestinal varices is present in individuals with the *NKX2‐3* variant. Two individuals (III:4 and VI:1) were found to have normal spleens on abdominal ultrasound imaging, but other individuals with intestinal varices did not have an assessment of the spleen. None of the individuals had other signs of varicosis or angiodysplasia such as epistaxis, lung, or other organ involvement, or varicosis of the legs.

## 4 DISCUSSION

Here, we report the first genetic cause for idiopathic intestinal varices, that is, mutations in either the *RPSA* gene (in four unrelated families) or the *NKX2‐3* gene (in an extended four‐generation pedigree). The *RPSA* gene encodes the SA ribosomal protein, which has myriad functions, among which are laminin‐binding, ribosomal functions, nuclear functions, modification of the extracellular matrix, and signal transduction (DiGiacomo & Meruelo, 2016). Our study establishes *RPSA* mutations as an important cause of intestinal varices and expands the clinical phenotypic spectrum associated with this gene. Previous work showed that *RPSA* mutations cause congenital asplenia with variable penetrance in humans (Boland et al., 2014; Bolze et al., 2018). Asplenia was also part of the extended phenotype in families described here. Both asplenia and intestinal varices may be occult for many years. Nonetheless, both disorders may have severe consequences, with asplenic patients developing meningitis or other forms of severe septic infections, and intestinal varices sometimes leading to severe bleeding necessitating hospitalizations, transfusions, and in some the removal of sections of the intestine. Several persons with *RPSA* mutation in these pedigrees developed bacterial meningitis or pneumonia, suggesting that persons with intestinal varices should be examined for the presence of a functional spleen.

A synthetic peptide recapitulating amino acids 161–180 of the *RPSA*‐encoded laminin‐receptor protein binds to laminin with high affinity (Castronovo, Taraboletti, & Sobel, 1991). Others have argued based on the crystal structure of the laminin‐receptor protein that of this putative laminin‐binding domain, only amino acid R180 is solvent‐exposed, with a critical role for a binding face involving Phe‐32, Glu‐35, and Arg‐155 (DiGiacomo & Meruelo, 2016; Jamieson, Hubbard, & Meruelo, 2011; Jamieson, Wu, Hubbard, & Meruelo, 2008). However, as far as we know, requirement specifically of the Arg‐180 residue for laminin‐binding properties of *RPSA* has not been experimentally proven, and Griffin et al. (2018) showed that this residue is required for pre‐rRNA processing. It is, therefore, unclear at the moment which cellular process exerted by NKX2‐3 are at the basis of asplenia pathogenesis. It is striking, however, that Arg‐180 and the adjacent Gln‐181 appear to constitute a hotspot for mutations leading to asplenia and intestinal varices (Bolze et al., 2018; Figure 3).

The *NKX2‐3* gene belongs to the NKX class of homeobox genes, which are key regulators of spleen ontogeny in embryogenesis (Brendolan et al., 2005; Brendolan, Rosado, Carsetti, Selleri, & Dear, 2007). *NKX2‐3* is closely related to the *NKX2‐5* gene, which plays a role in cardiogenesis, and spleen development (Koss et al., 2012). NKX2‐3 functions in development and function of the intestinal lymphoid system and intestinal vasculature (Kellermayer et al., 2016; Yu et al., 2011). *Nkx2‐3* is embryonically and postnatally expressed in the midgut and hindgut of the mouse and the chicken (Pabst, Schneider, Brand, & Arnold, 1997; Wang et al., 2000). High human NKX2‐3 messenger RNA (mRNA) expression is restricted to the colon, ileum, and spleen (gtexportal.org/home/gene/NKX2‐3; Pabst et al., 1997). *NKX2‐3* has hitherto not been linked to a genetic disorder but the gene is a strong candidate gene for intestinal varices as it is expressed in human intestinal microvascular cells where it regulates VEGFA and MADCAM‐1 signalling (Wang et al., 2000; Yu et al., 2011). Wang et al. (2000) replaced the NK‐2‐specific domain of *Nkx2‐3* with a LacZ construct in mice. They noted positive staining for LacZ in pharyngeal and visceral regions, including the vascular smooth muscle and endothelial cells of capillaries and small blood vessels of the intestine. Many of the *Nkx2‐3* ^lacZ∆HD^ homozygous mice died in the first weeks after birth, due to intestinal malabsorption. A striking observation was the presence of blood in the intestinal lumen. Mice who survived the neonatal period recovered and were apparently normal. Pabst, Zweigerdt, and Arnold, (1999) studied *Nkx2‐3‐*null mice generated by targeted gene disruption. Homozygous *Nkx2‐3* ^−/−^ mice were growth‐retarded, and the majority died before 3 weeks after birth. Reduced proliferation of the epithelium was shown in the fetal small intestine. In adult homozygous knockout mice, the small intestine showed altered villus morphology and increased villus size. Extensive extra vascularization of the small intestine was noted in these mice. Remarkably, the intestinal changes in *NKX2‐3* knockout mice were limited to the jejunum and ileum and absent in the colon, even though *Nkx2‐3* is also expressed in the hindgut. Outside of the digestive tract, splenic malformations were noted, with absent spleens in 20% of *Nkx2‐3* ^−/−^ mice and abnormalities in size or morphology of the spleen in the other mice (Pabst et al., 1999). Another study found ectopic vessel formation in the spleen of *Nkx2‐3* mutant mice, which were described as “high‐endothelial venule‐like” (Kellermayer et al., 2016).

Currently, no direct molecular connection between NKX2‐3‐ and RPSA‐related pathways is known, but data reviewed here provide hints that both genes may be involved in the same molecular processes in spleen and intestinal vasculature development from mesenchymal tissue during embryogenesis. An anatomical relationship may exist between asplenia and vascular malformation as the former is associated with anomalous venous drainage and possibly subsequent arteriovenous malformation (Arnautovic, Mazhar, Tereziu, & Gupta, 2017). Our finding that mutation of both *RPSA* and of *NKX2‐3* can cause intestinal varices complements previous studies showing that RPSA mutations affect spleen development in humans, and that knockout of *NKX2‐3* disrupts spleen development in mice. It is currently unclear why asplenia is not a feature associated with *NKX2‐3* mutations in humans. Possibly, the expression of the spleen phenotype may be variably penetrant as has been described for *RPSA* mutations (Bolze et al., 2018) and the condition may be occult. The identification of more patients with *NKX2‐3* mutations in the future may define a broader clinical phenotypic spectrum linked to NKX2‐3 mutations.

A possible molecular link between RPSA and NKX2‐3 could be through NKX2‐5, as NKX2‐3 and NKX2‐5 can heterodimerize (Kasahara et al., 2001) and NKX2‐5 is part of a functional module that contributes to the development of the spleen in mouse (Burn et al., 2008; Czompoly et al., 2011; Koss et al., 2012). Moreover, depletion of RPSA in *Xenopus* causes severe reduction of NKX2‐5 mRNA expression, that can be rescued by WT human RPSA mRNA but not by mutant p.Arg180Gly RPSA mRNA (Griffin et al., 2018). Strikingly, a frameshift variant in the *NKX2‐5* gene was found in a sporadic patient with asplenia and heart defects (Izumi, Noon, Wilkens, & Krantz, 2014; Koss et al., 2012).

In summary, we find that mutations in at least two genes can cause familial idiopathic intestinal varices, and hypothesize that there may be links between the molecular pathways involved in the development of the spleen and of the intestinal vasculature.

## Supporting information

Supporting information

## Data Availability

The data that support the findings of this study are available from the corresponding author upon reasonable request.
