# Supplementary material for: Mutations in RPSA and NKX2‐3 link development of the spleen and intestinal vasculature
Source: Hum Mutat. 2019 Sep 23;41(1):196–202. doi: 10.1002/humu.23909 (PMC6972609; doi:10.1002/humu.23909)
Supplement: Supplementary file 1 — Supporting information [file HUMU-41-196-s001.docx]

**SUPPLEMENTARY METHODS AND RESULTS**

**Clinical Reports:**

Family 1

The index patient is a 54 years old Dutch male with idiopathic intestinal varices in the colon and to a lesser extent in the small bowel. A case report of this family was published over 20 years ago (el-Dosoky et al., 1994) . The patient presented at the age of 10 years with anaemia. At the age of 16 years he experienced the first clinical bleeding with low haemoglobin concentration and melena. He has had numerous episodes of melena for which he required blood transfusions. He received his last transfusion at age 28 years. He has been treated with Thalidomide for a period two years with an apparent reduction of the frequency and intensity of the bleeding. At 48 years of age, he developed bacterial meningitis. Subsequent imaging showed asplenia. Family history revealed that his father was also known with intestinal bleeding due to colonic varices.

Family 2

The index patient is a 34 years old woman from Norway who suffered from recurrent episodes of anaemia due to several episodes with gastrointestinal bleedings for which she was hospitalized multiple times. It was difficult to establish the bleeding locus and several gastroscopies/colonoscopies were normal. With capsule endoscopy “some varicose veins/prominent venous structures” in the middle part of the jejunum and the distal part of ileum were observed, although none of them showed signs of active bleeding. On abdominal contrast CT a conspicuously twisty vein along a segment of the ileum, and two more suspicious vessels along a segment of the jejunum and in the wall of the proximal ascending colon were described. In 2013 they found a presumed bleeding source: bleeding varicose veins in the ascending colon and right part of the transverse colon. After the onset of gastrointestinal bleeding in 2010 extensive imaging was performed. The spleen was described to be “multiseptated” or ”fragmented”, probably representing polysplenia. Furthermore, the patient had recurrent tonsillitis and upper airway infections during childhood and adolescence. At age the age of three years she was hospitalized with bacterial meningitis. Tonsillectomy was performed at the age of 4 years. Due to recurrent and later chronic middle ear infections she was repeatedly treated with ear tubes. She developed a cholesteatoma on the right side and was surgically treated for that in 2010, 2011 and 2012.

Her 71 years old father has had recurrent episodes with anaemia since early childhood. At the age of 12 he was hospitalized due to dizziness, black stools and severe anemia and was treated with blood transfusions. During the years he has had a chronic tendency to anemia in spite of continuing oral iron therapy, with intermittent hospitalizations due to exacerbation of symptoms. Several years usually passed between episodes. He has been extensively investigated throughout, without a clear bleeding locus on gastroscopy or colonoscopy and with normal duodenal biopsies. On colonoscopy from 1998 they commented on a prominent vein in the area of the right colonic flexure and also in the recto sigmoid area. A capsule endoscopy performed in 2011 demonstrated areas with erythema in the proximal duodenum without ongoing bleeding. Supplementary gastroscopy showed a swollen duodenum and red spots resembling small angiodysplasias. In the fundus and esophagus small varices were observed. He does not have the same susceptibility to infections as his daughter, although he has asplenia. It is presumed that he has the same condition as his daughter. There are no other known family members with a similar clinical phenotype.

Family 3

This family has been reported previously as presenting idiopathic congenital asplenia (Bolze et al., 2013). Patient II.3, is a 48-year-old male who initially presented at an age of 17-years old with severe fatigue and anaemia with an Hb of 3g/l. His anaemia was managed for 5 years with repeated transfusions as no cause could be identified at that time. CT scan investigations for a GI cause of blood loss identified asplenia. Aged 24-years-old he underwent an exploratory laparotomy which identified abnormal dilated vessels in the wall of the distal duodenum. Since then the patient has undergone repeated argon laser cauterisation of duodenal blood vessels approximately every 6 months, with long-term oral iron suppletion. His mother (individual I.2) also has a history of intestinal angiodysplasia, and anaemia, which has necessitated laser treatment. Faecal occult blood tests of the younger generation were all negative. They have not had GI endoscopy as there has not been any clinical indication for this so far.

Family 4

The clinical history of this family has been reported previously (Wurfel et al., 2011). The index patient is a 16 years old girl, the second child of nonconsanguineous Caucasian parents. She developed iron-deficiency anaemia treated by erythrocyte transfusion from the age of 2 onwards. At preschool age, melena was observed once, but no underlying pathology was found. At the age of 11 years she was hospitalized with iron-deficiency anaemia and received iron substitution therapy with only transient effectiveness. At 16 years of age, she was again admitted for evaluation of severe iron-deficiency anaemia. Fecal occult blood testing was positive on multiple occasions, but esophagogastroduodenoscopy and colonoscopy failed to show the source of the bleeding. High-definition television gastroscopy revealed distorted teleangiectatic vessels in the stomach and numerous angiodysplastic lesions in the duodenum. In addition, capsule endoscopy detected multiple small angiodysplastic lesions in the duodenum and jejunum. Doppler ultrasound and magnetic resonance imaging with angiography of the abdomen confirmed asplenia but did not detect further vascular malformations.

The duodenal lesions were treated by 5 cycles of argon plasma coagulation through a double-balloon enteroscopy. This confirmed the findings of the capsule endoscopy and demonstrated multiple areas with polypoid mucosal lesions and purplish colored angiectasia in the jejunum. Pathological vascular patterns in the mucosa and submucosa of the stomach with dilated mucosal capillaries and abnormal submucosal arterioles were diagnosed in bioptic specimens. Biopsies of the jejunum revealed only dilated capillaries in the mucosa, possibly compatible with the diagnosis of angiodysplasia while the submucosa was not represented in the biopsies.

Family 5

The index patient of family 5 is a young Dutch girl (IV:1) with recurrent episodes of rectal bleeding. She underwent colonoscopy and upper gastrointestinal endoscopy for the first time at age 7 years. Colonoscopy showed no significant abnormalities, apart from some distended blood vessels. Colon biopsies suggested spirochetosis, treated with antibiotics. Upper gastrointestinal endoscopy was completely normal. However, rectal bleeding kept recurring. At age 10 years, she underwent a second colonoscopy. Prominent, distended blood vessels were now seen in the sigmoid (see figure 2). Colon biopsies showed limited nonspecific chronic inflammation. At 11 years of age, a massive bleeding occurred with a drop in haemoglobin concentration to 4.3 mmol/l, requiring transfusion of packed red blood cells. Again, colonoscopy was performed, which showed blood clots in the colon and very fragile colonic mucosa. However, the bleeding locus could not be identified. Extensive work-up by different imaging modalities (CTA, abdominal MRI, small bowel MRI and abdominal ultrasound) did not show thrombosis of the superior mesenteric vein or portal vein. Signs of portal hypertension were not found. Prominent vascular structures were noticed in the wall of the sigmoid, thought to be colonic varices . The patient’s mother (patient III:4 in figure 1) was already known to have varicosis coli. This was discovered as an incidental finding at age 32 years during a screening colonoscopy performed because of a positive family history for colon cancer.

The girl and her mother were referred to the genetics clinic. The pedigree revealed multiple family members with intestinal varices. The mother’s brother (patient III:5) was diagnosed at the age of 19 with a metastatic, moderately differentiated and necrotic adenocarcinoma, mostly likely originating from his colon. He died a few months after the diagnosis. It could not be retrieved whether colonoscopy was done and whether or not colonic varices were present.
Patient III:8 is a 29 year old woman, also known with blood vessel anomalies of the colon. At the age of 26 she underwent a colonoscopy to rule out inflammatory bowel disease because of complaints of chronic abdominal pain and altered bowel habits. Distended venous blood vessels were noted in the ileum and colon. Upper gastrointestinal endoscopy showed no abnormalities. She has no history of significant rectal bleeding. Her brother (III:7) experienced some rectal bleeding in the past, but never underwent a colonoscopy. Her father (II:12) has no history of rectal bleeding and he has never had a colonoscopy.

Patient III:9 is a, a 38 years old man. At the age of 33 he underwent a colonoscopy because of an episode of severe rectal bleeding and because of the family history of varicosis coli. Mild varicosis was seen in his colon. In addition, three adenomatous polyps were resected. Surveillance colonoscopy was performed three years later. No polyps were detected and the mucosa was described as oedematous with varicosis coli from cecum to sigmoid. Rectal bleeding occurred monthly until addition of a bulk-forming agent to the diet.

The mother of patient III:9 (individual II:15) is also known with intestinal varices and rectal bleeding at the age 0f 56. In addition, the sister of patient III:9 (individual III:10) also underwent a colonoscopy because of rectal bleeding which confirmed the presence of varicosis coli.

***Supp. Table S1.*** *Primer sequences used for Sanger sequencing of NKX-3 and RPSA. Lower case nucleotides indicate M13 sequence, upper case nucleotides indicate gene-specific sequence. F: forward primer, R: reverse primer.*

| **gene** | **direction** | **Exon** | **sequence 5' to 3'** |
| --- | --- | --- | --- |
| NKX2-3 | F | 1 | tgtaaaacgacggccagtGAGTCCAGGAGGAGAGC |
| NKX2-3 | R | 1 | caggaaacagctatgaccCCCCAGCCAAAGGGTAAA |
| NKX2-3 | F | 2 | tgtaaaacgacggccagtGTCACGGTGCTCCAGGA |
| NKX2-3 | R | 2 | caggaaacagctatgaccGGGAAGCTGTTGTAGGAGTAG |
| NKX2-3 | F | 3 | tgtaaaacgacggccagtAGTCTCTGGAGCTTGGC |
| NKX2-3 | R | 3 | caggaaacagctatgaccCCCTTCTCGAGCTTCAGTC |
|  |  |  |  |
| RSPA | F | 2 | tgtaaaacgacggccagtTTTAAGGGTGTGCCCTACT |
| RSPA | R | 2 | caggaaacagctatgaccGGTCTGGCTTCATTTAAGGAAGATA |
| RSPA | F | 3 | tgtaaaacgacggccagtCCAGTAGAGCTTGCCTCTATGA |
| RSPA | R | 3 | caggaaacagctatgaccTGACCTCGTAATACGCCG |
| RSPA | F | 4 | tgtaaaacgacggccagtTGCTTTACATGGAGTAGTAGTGAT |
| RSPA | R | 4 | caggaaacagctatgaccGGCCAGTCAGTAGCCTCAA |
| RSPA | F | 5 | tgtaaaacgacggccagtACAATTGCTTTTCAACTAAGAGATG |
| RSPA | R | 5 | caggaaacagctatgaccGGAAAGACTTACAGGGACTGAC |
| RSPA | F | 6 | tgtaaaacgacggccagtCTTGTGGTTACATAAGCAAATTGG |
| RSPA | R | 6 | caggaaacagctatgaccGGAATGAAAACCTCTTAAAGCTAGT |
| RSPA | F | 7 | tgtaaaacgacggccagtCAGTGTGCTACAGCATCTGATA |
| RSPA | R | 7 | caggaaacagctatgaccCCAGGTATTCTGATCTGGAGTAA |

***Supp. Table S2.*** *Shared exome sequencing variants between two distantly related affected individuals from family 5 (individuals IV-1 and III-9) leading to identification of NKX2-3 as varices-associated gene in this family.*

| **Position (hg37)** | **Reference** | | **Variant** | **Gene** | **Reference transcript** | **mRNA effect** | **Protein effect** |
| --- | --- | --- | --- | --- | --- | --- | --- |
| Chr17:9536215 | C | T | | CFAP52 | NM_145054.4 | c.1185C>T | p.(Asp395=) |
| Chr7:30694422 | G | C | | CRHR2 | NM_001202483.1 | c.1098C>G | p.(Ser366Arg) |
| Chr3:186256716 | C | T | | CRYGS | NM_017541.2 | c.306G>A | p.(Gly102=) |
| Chr10:96824658 | C | T | | CYP2C8 | NM_000770.3 | c.541G>A | p.(Val181Ile) |
| Chr17:9725122 | A | G | | GSG1L2 | NM_001310219.1 | c.267T>C | p.(His89=) |
| Chr10:88441336 | C | T | | LDB3 | NM_001171610.1 | c.465C>T | p.(Leu155=) |
| Chr10:101293153 | C | - | | NKX2-3 | NM_145285.2 | c.268del | p.(Gln90Argfs*25) |
| Chr17:4714225 | G | A | | PLD2 | NM_002663.4 | c.989G>A | p.(Arg330Gln) |
| Chr10:99969085 | C | G | | R3HCC1L | NM_001256619.1 | c.1214C>G | p.(Thr405Ser) |
| Chr3:133654649 | C | T | | SLCO2A1 | NM_005630.2 | c.1783G>A | p.(Ala595Thr) |
| Chr12:64202670 | A | G | | RXYLT1 | NM_014254.2 | c.1130A>G | p.(Lys377Arg) |
| Chr3:133331277 | G | A | | TOPBP1 | NM_007027.3 | c.3991C>T | p.(Arg1331Cys) |
